# Radiotherapy in lymphoepithelioma-like carcinoma of the skin: review of the literature and report of a recurrent and metastatic case

**DOI:** 10.1007/s00066-019-01516-8

**Published:** 2019-09-09

**Authors:** A. Mucha-Małecka, K. Urbanek, A. Ambicka, P. Majchrzak, K. Małecki

**Affiliations:** 1 Clinic of Oncology and Department of Radiotherapy, Maria Skłodowska-Curie Memorial Cancer Center and Institute of Oncology, Cracow Branch, Garncarska 11, 31–115 Cracow, Poland; 2 Department of Pathology, Maria Skłodowska-Curie Memorial Cancer Center and Institute of Oncology, Cracow Branch, Garncarska 11, 31–115 Cracow, Poland; 3 Department of Radiotherapy for Children and Adults, University Children’s Hospital of Cracow, Wielicka 265, 30–663 Cracow, Poland

**Keywords:** Lymphoepithelioma-like carcinoma of the skin, Surgery, Radiotherapy, Chemoradiation, Lymph node metastases, Primäres lymphoepitheliomähnliches Karzinom der Haut, Chirurgie, Strahlentherapie, Radiochemotherapie, Lymphknotenmetastasen

## Abstract

Primary lymphoepithelioma-like carcinoma of the skin (LELCS) is a very rare cutaneous neoplasm. Only about 70 cases have been documented in the literature. There are no prospective data regarding treatment methods. Surgical excision is sufficient therapy in the majority of cases. Radiation therapy is sometimes used in case of recurrence or positive margins after surgery. The metastatic potential of LELCS is extremely low and only five previously documented cases of lymph node spread have been reported. We present the case of an 80-year-old male with a tumor primarily located on the lower eyelid, with two regional recurrences and cervical lymph node spread after surgery, treated with concurrent chemoradiation. According to the available data, this is the first case of lymph node spread from an eyelid location and the first nodal recurrence after surgery.

## Zusammenfassung

Das primäre lymphoepitheliomähnliche Karzinom der Haut („primary lymphoepithelioma-like carcinoma of the skin“, LELCS) ist ein sehr seltener Hauttumor. In der Literatur wurden bisher nur ungefähr 70 Fälle beschrieben. Prospektive Studien, insbesondere bezüglich Therapie, fehlen. In den meisten Fällen ist die alleinige chirurgische Exzision ausreichend. Eine Strahlentherapie wird gelegentlich bei rezidivierten Tumoren oder im Fall positiver bzw. knapper Resektionsgrenzen eingesetzt. Das Metastasierungspotenzial des LELCS ist gering. Nur 5 Fälle einer lokoregionären lymphogenen Metastasierung wurden dokumentiert. Die Autoren berichten über einen 80-jährigen Mann, der ein lymphoepitheliomähnliches Karzinom am Unterlid des Auges entwickelte und 2 lokale bzw. lokoregionäre Rezidive erlitt. Bei lokalem und lokoregionärem Lymphknotenbefall wurde der Patient simultan radiochemotherapiert. Nach Informationen der Autoren ist das der erste beschriebene Fall der Ausbreitung eines LELCS-Lidtumors in die lokoregionären Lymphknoten und der erste Fall eines Lymphknotenrezidivs nach der Operation.

## Introduction

Primary lymphoepithelioma-like carcinoma of the skin (LELCS) is a very rare cutaneous neoplasm. The first case was described by Swanson et al. in 1988 [1], and only about 70 cases have been documented since that time [2]. The tumor morphology bears numerous similarities to undifferentiated nasopharyngeal carcinoma; thus, every patient requires diagnostic assessment in order to exclude a metastatic character of the lesion. Surgical excision is the first-line treatment [3], and radiation therapy is also sometimes used in case of recurrence or positive margins after surgery. LELCS has an exceedingly low metastatic potential. There have been only five previously documented cases of lymph node spread [4–7], two of them originating from the head and neck region [6, 7]. We report the case of an 80-year-old male with a tumor primarily located on the lower eyelid, with two regional recurrences and a cervical lymph node spread after surgery, treated with concurrent chemoradiation. According to available data, this is the first case of lymph node spread from an eyelid location [8, 9], and the first nodal recurrence after surgery [10].

## Case presentation

**Fig. 1:**
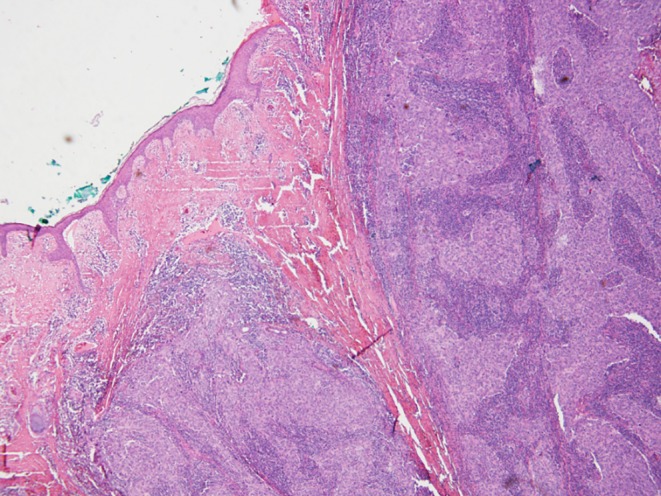
Lymphoepithelioma-like carcinoma of the skin infiltrating the dermis and leaving the epidermis uninvolved (hematoxylin and eosin, original magnification 40x)

**Fig. 2:**
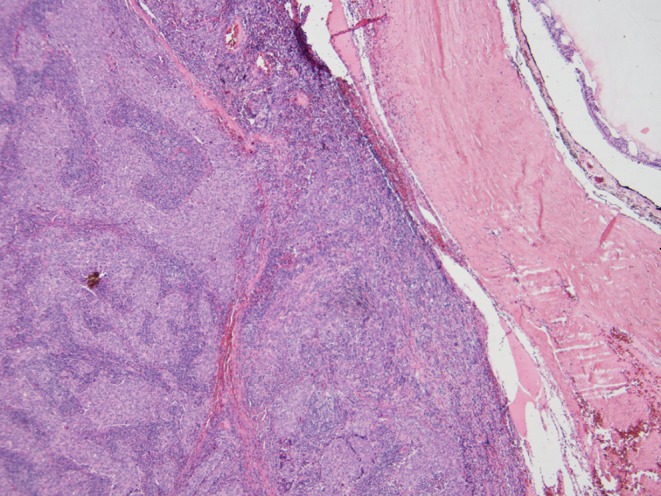
Lymphoepithelioma-like carcinoma of the skin compressing the globe (hematoxylin and eosin, original magnification 40x)

**Fig. 3:**
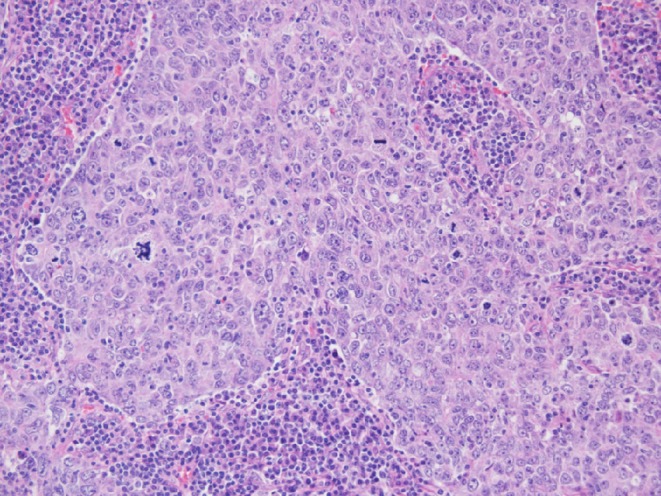
High-power view showing highly pleomorphic tumor cells with conspicuous mitotic activity and accompanying dense lymphoplasmacytic infiltrate (hematoxylin and eosin, original magnification 200x)

**Fig. 4:**
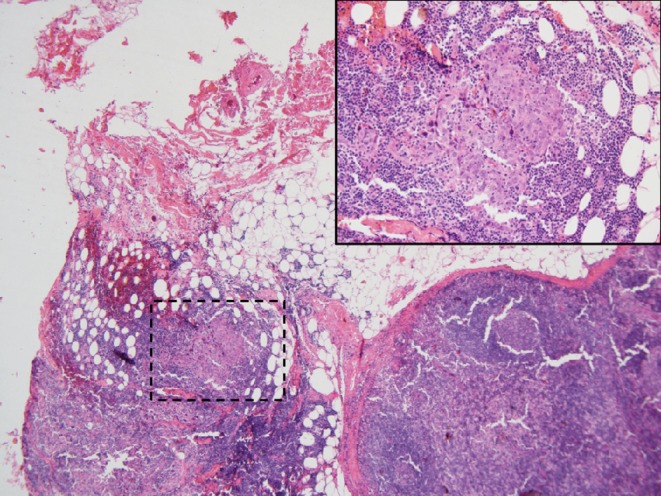
Cervical lymph node metastasis of lymphoepithelioma-like carcinoma of the skin (hematoxylin and eosin [HE], original magnification 40x; area outlined by *dashed line* is magnified in right upper corner; HE, original magnification 200x)

**Fig. 5:**
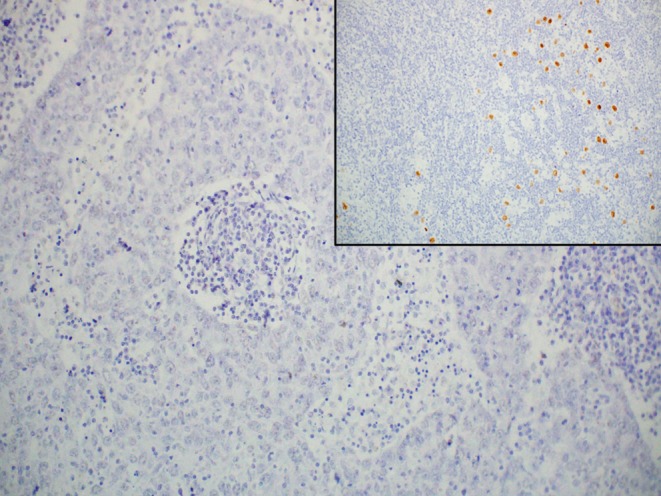
Negative EBER status in lymphoepithelioma-like carcinoma of the skin assessed by CISH; inset: EBER-positive external control (original magnification 200x)

An 80-year-old man with chronic HBV hepatitis, treated with entecavir, presented to our clinic after surgical removal of a tumor located on the left lower eyelid. The pathology report gave a diagnosis of LELCS, resected with very close margins. Laryngeal examination and computed tomography scan of the head and neck did not reveal any abnormalities in the nasopharyngeal region. After 8 months of observation, the patient presented with a local relapse. A PET-CT scan revealed a 2.9 × 3.3 cm hypermetabolic tumor of the left eyelid, penetrating into the orbit and compressing the eyeball, with a standardized uptake value (SUV) of 16.2. What is more, several cervical group II lymph nodes on the same side were found to be suspicious, with SUV of 8.8. Enucleation and group II lymph node dissection were performed. Histopathological examination confirmed the recurrence of primary disease: a well-circumscribed tumor spreading in the dermis (Fig. 1) and compressing the globe (Fig. 2) was observed, composed of sheets of large pleomorphic epithelioid cells with eosinophilic cytoplasm and high-grade nuclei with prominent nucleoli, accompanied by an intense infiltrate of lymphocytes and plasma cells (Fig. 3). Numerous mitotic figures, including atypical forms, complemented the picture of the neoplasm. Upon microscopic examination, the resection margins were found to be positive (R1 resection). Additionally, one of the eight removed lymph nodes showed a metastatic deposit of carcinoma cells (Fig. 4). Before starting adjuvant treatment, the tissue material obtained from both surgeries was reassessed at the pathology department of our institution. The primary diagnosis of lymphoepithelioma-like carcinoma of the skin was confirmed. For the detection of Epstein–Barr virus encoded RNA (EBER) by chromogenic in situ hybridization (CISH), a digoxigenin-labeled oligonucleotide probe was used (ZytoFast EBV Probe, ZytoVision GmbH, Bremerhaven, Germany). The procedure was performed according to the instructions of the manufacturer. A formalin-fixed paraffin-embedded tissue section of known nuclear EBER positivity was used as an external control. In the studied case, EBER status was found to be negative (Fig. 5).

**Fig. 6:**
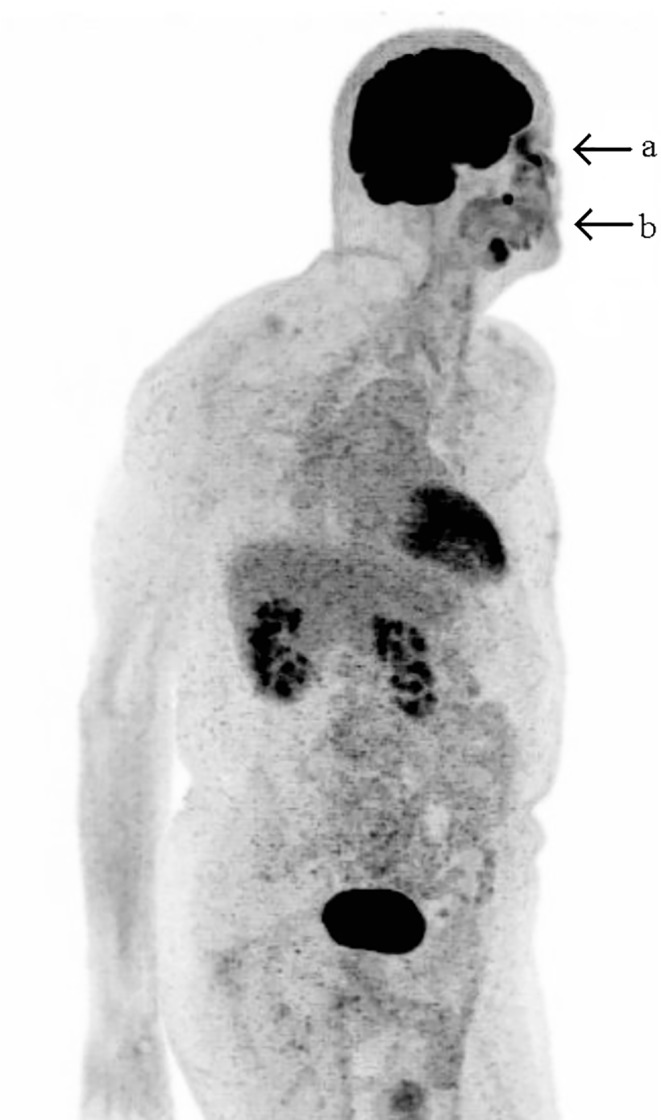
PET-CT showing recurrence in the orbit (*arrow a*) and two metastatic lymph nodes in unilateral groups Ib and IX (*arrow b*)

The patient qualified for adjuvant radiotherapy, which was planned after wound healing. Within 3 months after the surgery, the wound has healed and the patient presented with a suspicious, flesh-colored lesion located in the tumor bed. PET-CT confirmed progression in the orbit (SUV of 7.6) and two metastatic lymph nodes in unilateral groups Ib and IX (SUV of 8.7; Fig. 6). Due to the very short time from the surgery and problems with wound healing after previous surgery, the patient qualified for radiotherapy with concurrent chemotherapy. He received 70 Gy in 35 fractions to the orbit and metastatic lymph nodes; 63 Gy in 35 fractions to ipsilateral lymph node groups Ib, II, VIII, and IX; and 56 Gy in 35 fractions to elective ipsilateral lymph node groups: III, IV, V, and contralateral groups Ib-IV using an intensity-modulated radiation therapy (IMRT) technique with a simultaneous integrated boost. Due to the aggressive course of tumor, good performance status, and no comorbidities other than HBV, it was decided to use carboplatin plus paclitaxel-based chemotherapy. The carboplatin dose was calculated every week by the area under the curve (AUC2) using the Cockcroft–Gault formula and paclitaxel dose was 50 mg/m^2^ in a weekly regimen. Overall treatment tolerance was good. Grade 3 skin toxicity was observed. Due to neutropenia, the patient received four of five planned cycles of chemotherapy. Complete clinical remission of visible lesions verified by the medical board occurred after 4 weeks of therapy. Unfortunately, the patient died from pulmonary embolism confirmed in computed tomography 2 weeks after finishing the treatment. An autopsy was not performed.

## Discussion

Lymphoepithelioma-like carcinoma of the skin is a very rare cutaneous neoplasm presumably of adnexal origin [12]. It was originally described by Swanson et al. in 1988 [1]. The majority of previously published cases were diagnosed in people over 50 years of age and were located within the skin of the head and neck region [1, 11, 12]. LELCS on the trunk, arms, and penis have also been reported [2, 4]. Microscopically, LELCS is located in the mid- and deep dermis, and looks like small non-encapsulated nodules or cords with dense lymphoid infiltrate, often with invasion of subcutaneous fat or muscle tissue [1, 13]. Due to the similarity to poorly differentiated nasopharyngeal carcinoma, imaging and detailed laryngological examination should be performed in all patients [13, 14]. The best method to distinguish between these two conditions is EBV testing, which is negative for lymphoepithelioma-like carcinoma of the skin, as it was in our case. Literature data indicate a relationship between LELC and EBV infection in such anatomical locations as the salivary glands, thymus, lung, and stomach. Because of that, metastases of LELC from other organs should also be ruled out [5, 15, 16].

Our patient was treated for HBV. There are reports in the literature that HBV and HCV infections may be an etiological factor for lymphoepithelioma-like hepatocellular carcinoma (LEL-HCC) [17, 18]. Imaging studies did not show any changes in the liver of our patient.

Differential diagnosis should also exclude skin metastasis of Merkel cell carcinoma, squamous cell carcinoma, and lymphoma [3, 10].

Since LELCS is such a rare neoplasm, there are no prospective data on treatment methods. Only series of cases and case reports have been published in the literature so far. Surgery alone is sufficient therapy in the majority of cases; however, especially in cases with positive margins or perineural invasion, adjuvant treatment is sometimes used. Such treatment should be started immediately after wound healing. Unfortunately, in our case, wound healing was very prolonged, which could be related to the advanced age of the patient. Although the patient was evaluated every 3 weeks, radiotherapy could only be started 3 months after surgery, when the wound finally healed. This long waiting time for radiotherapy was probably the reason for progression of this relatively aggressive tumor. Table 1 presents the results of application of radiotherapy alone and chemoradiation according to the available literature.

**Table 1:** Results of application of radiotherapy alone and chemoradiation according to available literature

Study	Clinical situation	Radiotherapy technique	Clinical outcome
Swanson et al. [1] (1988)	Adjuvant treatment after surgery	No data	No data
Walker et al. [11] (1990)	Adjuvant treatment after surgery—positive surgical margins	44 Gy kV	18 months of follow-up
Ortiz-Frutos et al. [12] (1993)	Recurrence 2 years after surgery	No data	No data
Takayasu et al. [6] (1996)	Adjuvant treatment after surgery—cervical lymph nodes metastases	No data	6 years of follow-up
Ahmadi et al. [14] (2001)	Metastatic or satellite mass in the orbit, followed by two wide excisions of the forehead lesion	Concurrent chemoradiotherapy—2 cycles of cisplatin and 3 cycles of cisplatin and 5‑fluorouracil; radiotherapy: 66 Gy in 33 fractions	No data
Gille et al. [13] (2012)	Adjuvant treatment after surgery—positive margins and perineural invasion	Concurrent chemoradiotherapy—5 cycles of cisplatin (20 mg/m^2^) and 60 Gy in 30 fractions to the tumor bed and to track the infra-orbital nerve	2 years of follow-up
Lassen et al. [3] (2014)	Adjuvant treatment after surgery—perineural invasion in re-excision	51 Gy in 17 fractions in the operated area with margin of 1 cm	2 years of follow-up

Apart from our case, adjuvant therapy was used in seven cases, with different radiotherapy doses and chemotherapy regimens. Regardless of clinical setting and indications for such treatment, none of the authors described relapse or severe complications after therapy. Due to the extremely rare incidence of LELCS, there will be no evidence-based management of this disease.

## Conclusion

Available data suggest that radiotherapy may be a safe and reasonable option for patients who have undergone surgical treatment and are in the group with a high risk of local relapse, especially those with positive margins. In patients with lymph node involvement, concurrent chemotherapy should be considered.

## References

[1] Swanson SA, Cooper PH, Mills SE, Wick MR (1988). Lymphoepithelioma-like carcinoma of the skin. Mod Pathol.

[2] Morteza Abedi S, Salama S, Alowami S (2013). Lymphoepithelioma-like carcinoma of the skin: case report and approach to surgical pathology sign out. Rare Tumors.

[3] Lassen CB, Lock-Andersen J (2014). Lymphoepithelioma-like carcinoma of the skin: a case with perineural invasion. Plast Reconstr Surg Glob Open.

[4] Hall G, Duncan A, Azurdia R, Leonard N (2006). Lymphoepithelioma-like carcinoma of the skin: a case with lymph node metastases at presentation. Am J Dermatopathol.

[5] Kazakov DV, Nemcova J, Mikyskova I, Michal M (2007). Absence of Epstein-Barr virus, human papillomavirus, and simian virus 40 in patients of central European origin with lymphoepithelioma-like carcinoma of the skin. Am J Dermatopathol.

[6] Takayasu S, Yoshiyama M, Kurata S, Terashi H (1996). Lymphoepithelioma-like carcinoma of the skin. J Dermatol.

[7] Lee J, Park J, Chang H (2015). Lymphoepithelioma-like carcinoma of the skin in the cheek with a malignant metastatic cervical lymph node. Arch Plast Surg.

[8] Maruyama M, Miyauchi S, Ohtsuka H, Miki Y (1995). Lymphoepithelioma-like carcinoma originating on the eyelid. J Dermatol.

[9] Ho W, Taylor A, Kemp E, Roberts F (2005). Lymphoepithelioma-like carcinoma of the eyelid: a report of two cases. Br J Ophthalmol.

[10] Welch PQ, Williams SB, Foss RD (2011). Lymphoepithelioma-like carcinoma of head and neck skin: a systematic analysis of 11 cases and review of literature. Oral Surg Oral Med Oral Pathol Oral Radiol Endod.

[11] Walker AN, Kent D, Mitchell AR (1990). Lymphoepithelioma-like carcinoma of the skin. J Am Acad Dermatol.

[12] Ortiz-Frutos FJ, Zarco C, Gil R, Ballestin C, Iglesias L (1993). Lymphoepithelioma-like carcinoma of the skin. Clin Exp Dermatol.

[13] Gille TM, Miles EF, Mitchell AO (2012). Lymphoepithelioma-like carcinoma of the skin treated with wide local excision and chemoradiation therapy: a case report and review of the literature. Case Rep Oncol Med.

[14] Ahmadi MA, Prieto VG, Clayman GL, Ginsberg LE, Esmaeli B (2001). Lymphoepitheliomalike Carcinoma of the orbit. Arch Ophthalmol.

[15] Iezzoni JC, Gaffey MJ, Weiss LM (1995). The role of Epstein-Barr virus in lymphoepithelioma-like carcinomas. Am J Clin Pathol.

[16] Gillum PS, Morgan MB, Naylor MF, Everett MA (1996). Absence of Epstein-Barr virus in lymphoepitheliomalike carcinoma of the skin. Polymerase chain reaction evidence and review of five cases. Am J Dermatopathol.

[17] Emile JF, Adam R, Sebagh M (2000). Hepatocellular carcinoma with lymphoid stroma: a tumour with good prognosis after liver transplantation. Histopathology.

[18] Wang JK, Jin YW, Hu HJ (2017). Lymphoepithelioma-like hepatocellular carcinoma. A case report and brief review of literature. Medicine.

